# Supplementary material for: Single Cell Effects of Photobiomodulation on Mitochondrial Membrane Potential and Reactive Oxygen Species Production in Human Adipose Mesenchymal Stem Cells
Source: Cells. 2022 Mar 11;11(6):972. doi: 10.3390/cells11060972 (PMC8946980; doi:10.3390/cells11060972)
Supplement: Supplementary file 1 [file cells-11-00972-s001.zip › cells-1571081-supplementary.pdf]

Single Cell Effects of Photobiomodulation on Mitochondrial Membrane Potential and Reactive Oxygen Species Production in Human Adipose Mesenchymal Stem Cells

Supplementary Material

Table S1. Time-lapse Rhodamine 123 probe in inducing ΔΨm in PBM

	**Fluence (J/cm^2)** [1]		
	**2.5**	**5**	**10**
**Area** (cm^2)		3×10^{-6}	
**Real power** (W)	0.076	0.076	0.076
**Time (s)**	10	20	40

[1] Fluence (J/cm^2) = Irradiance (W) x Time (s)/ Area (cm^2)

Table S2. Fluence-dependent effects in promoting ΔΨm in the irradiated-hADSCs

	**Fluence (J/cm^2)** [1]		
	**2.5**	**5**	**10**
**Area** (cm^2)		6×10^{-6}	
**Real power** (W)	0.076	0.076	0.076
**Time (s)**	5	10	20

[1] Fluence (J/cm^2) = Irradiance (W) x Time (s)/ Area (cm^2)

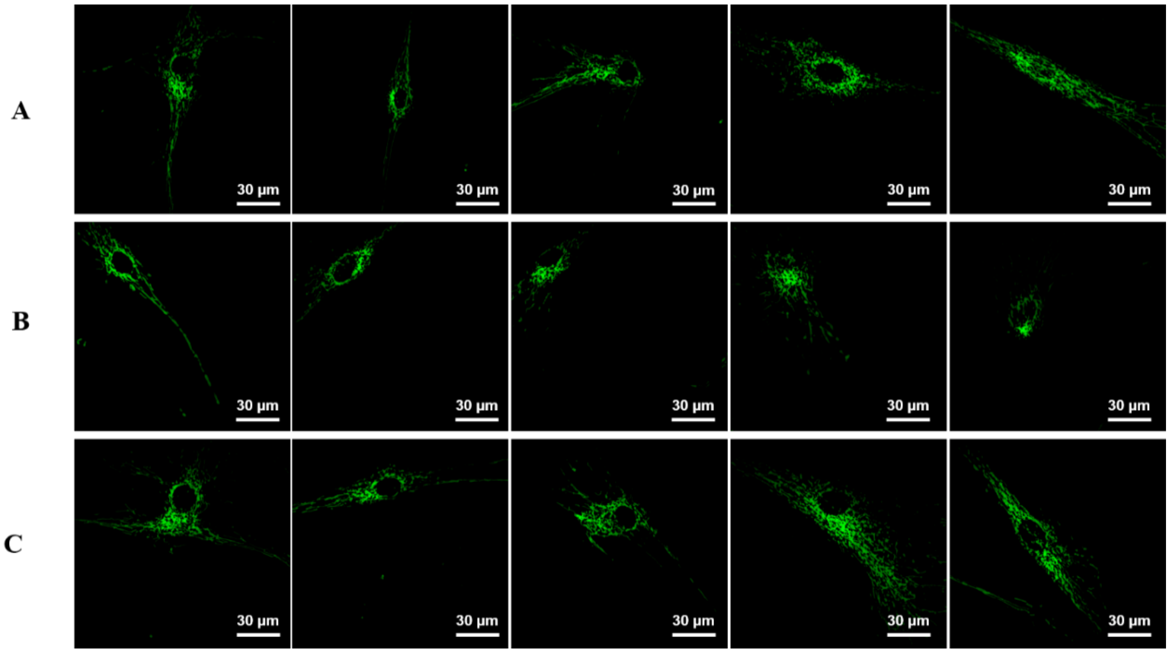

Figure S1. Representative fluorescence microscopy images of Rh123 probe in an investigation of various Rh123 concentrations in hADSCs. (A) Rh123's performance of 1.0 µM; (B) 5.0 µM, (C) 10 µM. These cells were automatically selected and captured after 30 mins staining by ORCA-Flash 4.0 V3 Digital CMOS camera.

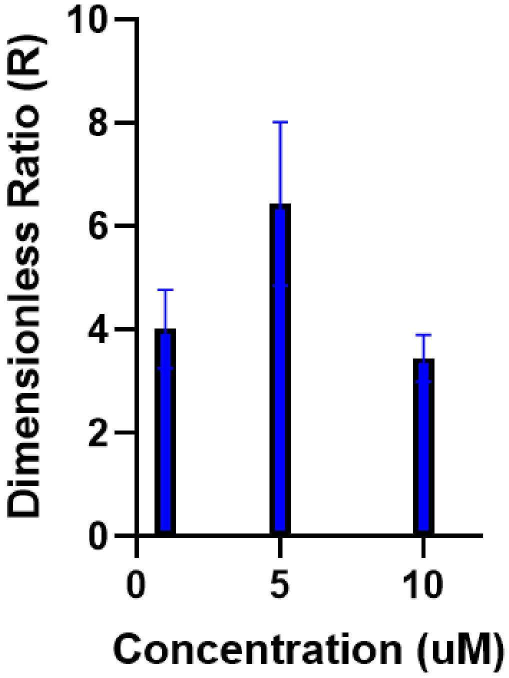

Figure S2. A plot of investigation of various Rh123 concentrations in hADSCs. Values were the mean dimensionless ratio ± std (n = 5). Statistical analysis (Pair t-test) were significant differences between the dimensionless ratio of Rh123's concentration at 1.0 µM and 5.0 µM; and 5.0 µM and 10 µM ($p \leq 0.05$), while that of Rh123's concentration at 1.0 µM and 10 µM was not.

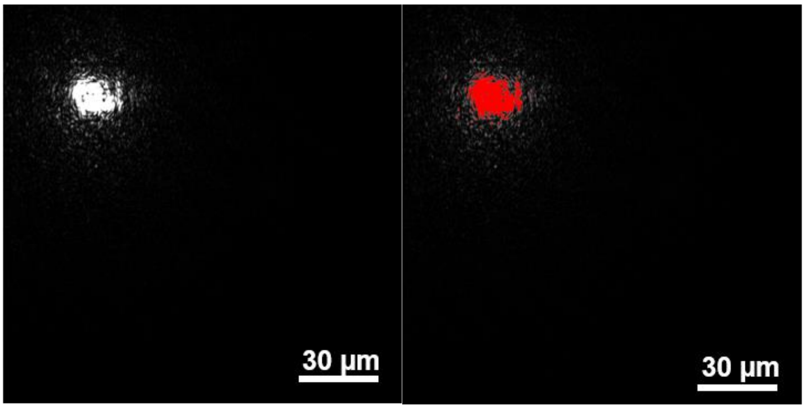

Figure S3: By setting up of a treatment zone that refers to the hADSCs size, the pulsed laser beams' spot area was created, and its area was then calculated by ImageJ software, 3×10^{-6} (cm^2) in this case (Figure S3). From that, a good range of fluences (2.5; 5; 10 J/cm^2) in PBM was then identified by changing different treatment times, including 10, 20, 40 seconds and keeping input power (20 mW) and area (3×10^{-6} µm^2) stable. In addition, a pass filter (OD = 4) was used to reduce the energy of a laser beam of wavelength 830 nm.

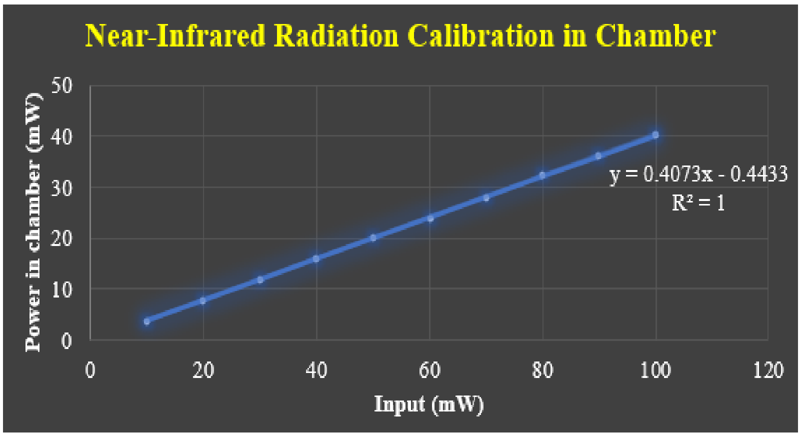

Figure S4: A wide range of fluency, which is used in photobiomodulation experiments, is calculated based on three main elements, including power (W), time (s), and area (cm^2). A formula (S1) is shown above:

$$\textbf{Fluence} = \frac{\textbf{Power (W)x Time (s)}}{\textbf{Area (cm}^2\textbf{)}} \ \textbf{(J/cm}^2\textbf{)} \qquad \text{(S1)}$$

For one, power (J), which refers to real power in the chamber, is quantified based on power input and transmission of Nikon microscope objectives, which is investigated and indicated only 59% transmission capacity of wavelength 830 nm in the previous studies. The results are calculated according to the formula (Power in chamber = power output x 100/59). An wide investigated range of power input is 10, 20, 30, 40, 50, 60, 70, 80, 90, 100 (mW) and

the real power in chamber is 3.64; 7.36; 11.73; 15.9; 19.95; 24.03; 28.12; 32.2;36.12; 40.25 (mW), respectively. The diagram illustrates that a straight line (y = 0.4073 × – 0.4433) means a continuous change in the values at different power input values, and an R-squared value equal to one indicated a slight significance in analysis processing (Figure S4).

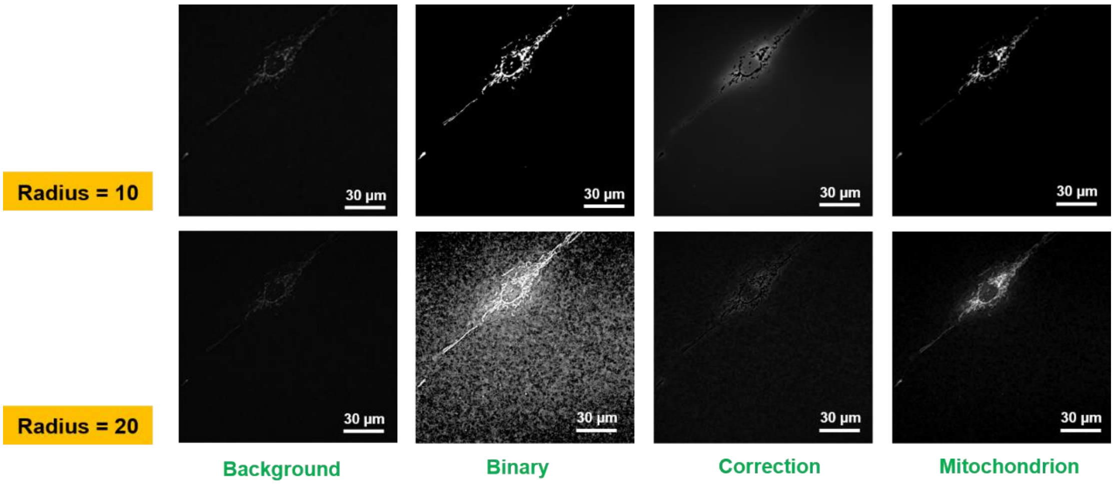

Figure S5: To enhance the precise calculation in mitochondrial membrane potential (ΔΨm), background noise elimination is necessary before measuring the mean fluorescence intensity of the Rh123 before and after PBM. In addition, the original mitochondrial structure was observed clearly without image issues by developing a code and inputting it into Matlab to remove background noise. Note that an appropriate radius is a primary key for eliminating background noise in these cases. Taking Figure S5 as an example, the radius of 20 is unsuitable due to the background noise that existed and was observed in the Mitochondrion image. In contrast, the mitochondrial structure was observed clearly with a radius of 10.

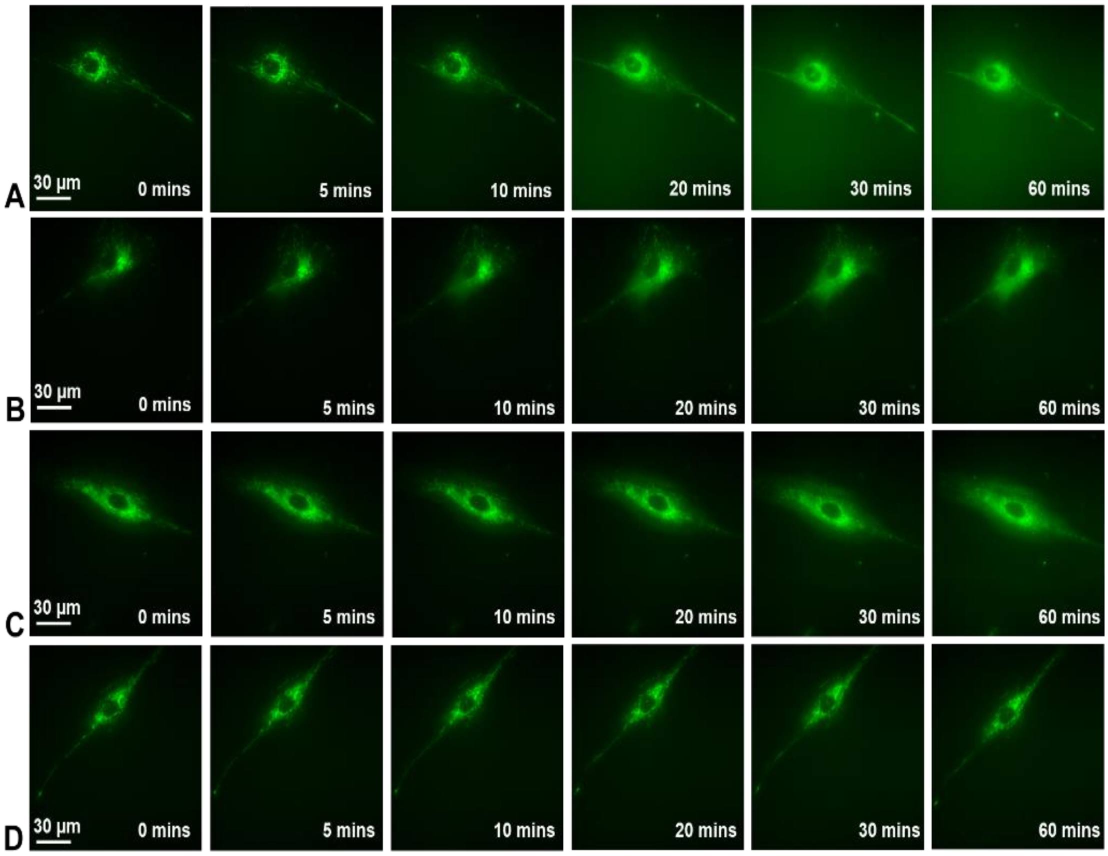

Figure S6. Representative time-lapse fluorescence microscopy images of Rh123 probe in PBM. (A) Control group (0 J/cm^2) and (B-D) Photobiomodulation group (2.5, 5, and 10 J/cm^2), these images were captured at different time points 0, 5, 10, 20, 30, and 60 minutes by ORCA-Flash 4.0 V3 Digital CMOS camera, respectively. The increase of the fluorescence signals of Rh123 probe illustrated mitochondrial membrane depolarization

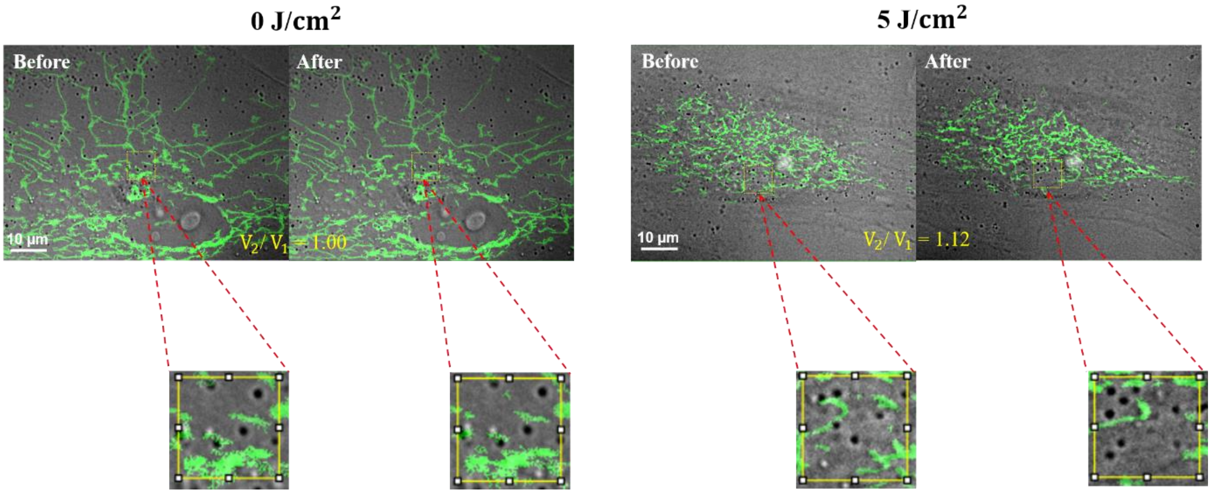

Figure S7. The emergence between bright field and fluorescence images in promoting the vesicle transport in the irradiated-hADSCs (Passage 10) after 30 minutes treatment at a fluence

of 5 (J/cm^2) compared to control 0 (J/cm^2). The ROI area (64 × 64 pixels) was identified by ImageJ, followed by cutting it and measuring the velocity using Matlab software.
